# Placenta-Derived Osteoprotegerin Is Required for Glucose Homeostasis in Gestational Diabetes Mellitus

**DOI:** 10.3389/fcell.2020.563509

**Published:** 2020-09-01

**Authors:** Binbin Huang, Wen Zhu, Huashan Zhao, Fa Zeng, Esther Wang, Hefei Wang, Jie Chen, Mengxia Li, Chen Huang, Lirong Ren, Jianmin Niu, Jian V. Zhang

**Affiliations:** ^1^Center for Energy Metabolism and Reproduction, Shenzhen Institutes of Advanced Technology, Chinese Academy of Sciences, Shenzhen, China; ^2^Shenzhen College of Advanced Technology, University of Chinese Academy of Sciences, Shenzhen, China; ^3^Department of Clinical Pharmacy and Translational Medicine, School of Pharmacy and Biomedicine, Shenzhen Institutes of Advanced Technology, Chinese Academy of Sciences, Shenzhen, China; ^4^Shenzhen Maternity and Child Healthcare Hospital, Southern Medical University, Shenzhen, China; ^5^Biological Sciences Collegiate Division, The University of Chicago, Chicago, IL, United States; ^6^Shenzhen Bao’an Traditional Medicine Hospital, Guangzhou University of Chinese Medicine, Shenzhen, China; ^7^Guangdong Key Laboratory of Nanomedicine, Shenzhen, China

**Keywords:** osteoprotegerin, GDM, embryo transplantation, placenta, β-cell

## Abstract

Osteoprotegerin (OPG) is involved in various biological processes, including bone remodeling, vascular calcification and pancreatic β-cell function. Although some clinical studies have shown an increase in serum OPG level during pregnancy, the role of OPG in gestational diabetes mellitus (GDM) is largely unknown. Therefore, we explored the effect of OPG in metabolic homeostasis during pregnancy. We initially evaluated serum OPG levels using ELISA and western blotting techniques on samples from GDM patients. We also assessed OPG expression levels in maternal mice. We then used blastocysts transduced with lentiviruses capable of trophoblast-specific transgene expression to establish placenta-specific OPG knockdown or overexpression mouse models for functional and mechanistic investigation after embryo transplantation. We found that OPG expression was positively associated with GDM in clinical samples, and OPG levels were significantly increased in GDM patient sera and term placenta. Serum OPG was significantly increased in maternal compared to non-pregnant mice, and expression levels of OPG were the highest in placenta compared with other organs, including bone, liver and pancreas. OPG was also significantly increased in pregnant mice fed a high-fat diet (HFD). Placenta-specific OPG knockdown induced glucose intolerance, decreased β-cell proliferation and decreased serum insulin levels, whereas placenta-specific OPG overexpression promoted glucose tolerance and enhanced β-cell proliferation, which increased serum insulin production and decreased fetal weight in HFD-feeding pregnant mice. Placenta-derived OPG (pl-OPG) regulated glucose homeostasis during pregnancy via enhancement of β-cell proliferation, which suggests a potential therapeutic application of OPG for GDM.

## Highlights

- Placenta-derived OPG promotes interaction between pancreas and placenta in GDM.
- Placenta-derived OPG regulates blood glucose homeostasis in mice during pregnancy.
- Placenta-derived OPG directly promotes proliferation of β-cell during pregnancy.

## Introduction

Gestational diabetes mellitus (GDM) is characterized by an impaired glucose tolerance that is first recognized during pregnancy using a 75-g oral glucose tolerance test (OGTT) at 24–28 weeks of gestation, according to the IADPSG criteria (International Association of Diabetes and Pregnancy Study Groups Consensus Panel, Metzger et al., 2010). GDM is also defined by disordered glucose metabolism, hyperglycemia, hyperinsulinemia and insulin resistance (Liang et al., 2010; Yao et al., 2017). The placenta produces hormones that play roles in fetal growth, but these hormones also affect maternal insulin levels, which causes insulin resistance and increased insulin secretion (Gauster et al., 2012). The global morbidity of GDM is approximately 10–15% of all pregnancies, and it shows an increasing trend (Li et al., 2016). The complications associated with GDM include macrosomia, increased perinatal mortality, increased risk of type 2 diabetes later in the mother’s life, and metabolic syndrome in GDM patients and their offspring (Shao et al., 2002; Bellamy et al., 2009).

Oxygen and nutrient transport are involved in maintaining metabolic homeostasis between the fetus and placenta during pregnancy (Garcia-Patterson et al., 2004; Qiao et al., 2015; Li et al., 2016). Many genes and hormones are involved in GDM via effects on β-cell expansion and function. Therefore, no particular factor may be singled out to explain the development of GDM. We previously reported that G protein-coupled receptor 1 (GPR1) was associated with GDM (Huang et al., 2019), and there are many reports on the pathogenic factors of GDM, including genes, lifestyle and environmental factors, which indicates that the developmental molecular mechanism of GDM remains poorly understood (Bo et al., 2001).

Osteoprotegerin (OPG) is a soluble, secreted glycoprotein that was first discovered in a cDNA sequence analysis of rat small intestines in 1997. It is a new member of the tumor necrosis factor receptor superfamily, and it functions include inhibition of the differentiation and maturation of osteoclasts, inducing apoptosis of osteoclasts, and increasing bone density (Simonet et al., 1997). OPG is expressed in healthy and disease states of various tissues, including bone, heart, blood vessels, kidneys, liver, spleen, thymus, lymph nodes, placenta, adipose tissue, and pancreas (Shen et al., 2012; Bernardi et al., 2016). Clinical studies reported increased serum OPG levels in patients with type 1 and type 2 diabetes mellitus (Browner et al., 2001; Knudsen et al., 2003; Galluzzi et al., 2005). OPG could regulate the proliferation of β-cells in diabetic, young and aged rodent models via prolactin (Kondegowda et al., 2015). These studies suggest that OPG is an active participant in the metabolic process. OPG knockout mice and LPS induced inflammatory mouse models showed significantly decreased insulin secretion and impaired glucose tolerance. Glucose homeostasis were significantly improved after exogenous OPG supplementation (Kuroda et al., 2016). OPG is significantly increased during pregnancy and rapidly decreased to pre-pregnancy levels after delivery in clinical studies, which is consistent with previous studies in mice (Yano et al., 2001; Naylor et al., 2003).

Given OPG is involved in metabolism and is generally expressed before and after delivery (Yano et al., 2001; Naylor et al., 2003), it may play a role in glucose metabolism during pregnancy. To reveal the role of OPG in metabolic homeostasis during pregnancy we firstly demonstrated OPG was associated with GDM in clinic. Next, we used mouse model to confirm this association, namely GDM mouse also showed significantly increase of OPG compared to normal pregnant mice. Further, we found that OPG derived from placenta as a secretory factor involved in metabolism homeostasis in GDM mice model that were induced by high-fat diet (HFD), and placenta-derived OPG (pl-OPG) could directly promote islet β-cell proliferation, thereby increasing serum insulin production in maternal mice.

## Materials and Methods

### Human Samples

All women in the study underwent a 75-g OGTT at 24–28 weeks of gestation to identify GDM and were diagnosed according to IADPSG criteria (plasma glucose: 0 h (fasting) ≥ 5.1 mmol/L;1 h ≥ 10.0 mmol/L; 2 h ≥ 8.5 mmol/L). Human serum in the middle and late trimesters and full-term placentas after delivery were collected from normal women and GDM patients in the Shenzhen Maternity and Child Healthcare Hospital. Placental tissues were collected within 15 min of delivery, immediately snap-frozen in liquid nitrogen, and stored at −80°C. Informed written consent was obtained from the participants. The Committees on the Use of Humans for Teaching and Research, Shenzhen Institutes of Advanced Technology, Chinese Academy of Sciences and Shenzhen Maternity and Child Healthcare Hospital, Southern Medical University, approved all research protocols associated with clinical samples.

### Animal Experiments

Virgin female 7- to 8-week-old ICR (CD-1) mice and fertile 10- to 15-week-old male mice were obtained from Vitalriver (Keaoxieli, China) and acclimated to housing conditions for 1 week at a temperature of 22.0 ± 1°C, humidity of 40–60% and a 12-h light/dark cycle with fresh food and water available *ad libitum*. Vasectomy was performed on a portion of the fertile male mice. Female mice were mated with fertile or vasectomized males to induce pregnancy or pseudopregnancy, respectively (GD1 = day of vaginal plug). Pregnant mice were divided into two groups: chow and HFD (45% kcal fat, 35% kcal carbohydrates and 20% kcal proteins, Research Diets, United States). Blastocysts were collected from pregnant mice via a uterine wash with M2 medium at GD4. Placenta-specific transgene expression was described previously (Fan et al., 2014). In brief, the zona pellucida was removed from the blastocysts, and the blastocysts were transduced with lentiviruses for trophoblast-specific transgene expression. These cells were transplanted into pseudopregnant mice at GD3. A lentivirus vector (pLV1-U6/GFP/PURO) expressing scramble sequence (SCR: CCTAAGGTTAAGTCGCCCTCT) was used as a control, and the mouse shRNA-OPG sequence (shOPG: GCCTCCTGCTAATTCAGAAAG) was used in the OPG knockdown feeding with chow diet. Lentivirus vector (plVx-CMV-ccDB-EF1a-EGFP) expressing GFP alone was used as a control, and co-expression of mouse OPG overexpression and GFP was used in the OPG expression feeding with HFD. Concentrations of Lentivirus (1.25 × 10^10^ particles/ml) under light mineral oil for different duration of 6 h. Transduced blastocysts were washed with M2 medium to remove extra viruses and transferred into GD3 pseudo-pregnant mice, and 6 blastocysts per side uterine. The Committee on the Use of Live Animals for Teaching and Research, Shenzhen Institutes of Advanced Technology, Chinese Academy of Sciences, approved all research protocols associated with animal experiments. All animal experimental methods were performed in accordance with the approved guidelines and regulations.

### Glucose Homeostasis and Serum Parameters

Intraperitoneal glucose tolerance tests (IPGTTs) were performed as described previously (Huang et al., 2019). Briefly, unrestrained mice were injected intraperitoneally with 2 g of D-(+)-glucose/kg body weight (anhydrous D-(+)-glucose, Sinopharm Chemical Reagent Co., Ltd., China) after fasting for 16 h, and blood glucose was sampled from the tail at 0, 15, 30, 60, and 120 min on GD16 using a glucose monitor (Roche). The intraperitoneal insulin tolerance test (IPITT) was performed in unrestrained mice that were fasted for 6 h and injected intraperitoneally with insulin (0.75 U/kg body weight) (Sigma-Aldrich), and blood glucose was sampled from the tail at 0, 15, 30, 60, and 120 min on GD17 using a glucose monitor (Roche). Fasting blood glucose (FBG) was also measured after fasting for 6 h. Blood was collected via retro-orbital bleeding on GD18. Blood was centrifuged at 3500 rcf and 4°C for 10 min, and serum was collected from the supernatant. Serum insulin levels were detected using insulin kits (Millipore, United States). Serum OPG levels were detected using OPG kits (R&D Systems, United States). Serum prolactin (PRL) levels were measured using commercial iodine-125 radioimmunoassay (RIA) kits (Beijing North Biotechnology Research Institute). The sensitivity of the progesterone and estradiol RIAs was 20 ng/ml. The intra-assay and interassay errors were <10 and <15%, respectively.

### Immunofluorescence

Tissues, including pancreas and placenta samples, were rapidly removed from the animals and fixed in Bonn’s liquid, dehydrated in alcohol, embedded in paraffin, and sectioned. Morphological examination was performed on 5-μm sections of paraffin-embedded tissue using immunofluorescence. The antigens were retrieved in citrate buffer using microwave heat. The tissues were incubated with rat anti-CK8 (DSHB, United States), rabbit anti-OPG, mouse anti-PCNA, and guinea pig anti-insulin primary antibodies (Abcam, United Kingdom) followed by incubation with anti-rabbit IgG Alexa 568, anti-guinea pig IgG Alexa 568, anti-rat IgG Alexa 488 and anti-mouse IgG Alexa 488 (Invitrogen, United States). The expression of OPG, CK8, proliferating cell nuclear antigen (PCNA) and insulin was determined by fluorescence microscopy.

### Western Blotting and Real-Time RT-PCR Analysis

Protein extraction and western blotting were performed as previously described (Qiao et al., 2015). Antibodies for OPG, glucose transport protein 3 (GLUT3) (Abcam, United Kingdom), phosphorylated (p)AKT/AKT (CST, United States), PCNA, GAPDH and β-actin (Sigma, United States) were used. Real-time qPCR was performed as previously described (Huang et al., 2018). Total RNA was isolated from mouse tissues or cells using RNA plus liquid kits (Takara, Japan) following the manufacturer’s protocol. RNA (0.5–2 μg) was reverse transcribed into cDNA using reverse transcription kits (Toyobo, Japan). Expression levels of several genes were determined by real-time RT-PCR using a Roche LightCycler^TM^ instrument (Roche) with SYBR Green (Toyobo) detection according to the manufacturer’s protocol. The following RT-PCR primers were used: mouse primer sequence of 18S rRNA (forward, GATCCCGGCTCTTAATATTCGAAT; reverse, GCCAGAGTCTCGTTCGTTATC); and OPG (forward, CCGAGTGTGTGAGTGTGAGG; reverse CCAGCTTGCACCACTCCAA).

### Islet Isolation and Culture

Mouse islets were isolated following the injection of collagenase P through the pancreatic duct as previously described (Garcia-Ocana et al., 2000). Islets were treated with recombinant OPG (R&D Systems, 459-MO-100, United States) without FBS for 24 h. Glucose-stimulated insulin secretion (GSIS) was performed in islets that were preincubated in glucose-free Krebs-Ringer buffer (KRB) (114 mM NaCl; 4.7 mM KCl; 1.2 mM KH2PO_4_; 1.16 mM MgSO_4_; 2.5 mM CaCl_2_; 25 mM NaHCO_3_; 20 mM HEPES; and 0.2% fatty acid-free BSA, pH 7.4) for 1 h, then incubated for 1 h in KRB containing glucose (2.5 or 16.7 mM). Insulin secretion into the KRB and insulin contents were measured using insulin ELISA (Millipore). Insulin levels were normalized to the number of islets.

### Cell Culture

HTR-8/SVneo cells were provided by Dr. Charles H. Graham, Queen’s University (Kingston, ON, Canada) and cultured in DMEM-F12 (Gibco, Grand Island, NY, United States) supplemented with 10% fetal bovine serum (FBS; Gibco). Cells were cultured as previously described (Huang et al., 2019). Stable overexpression of OPG in HTR-8/SVneo cells was established using a human OPG expression vector and Lipofectamine 2000 transfection reagent (Invitrogen, United States).

### Statistical Analysis

All data are presented as the means ± SEM. Statistically significant differences were confirmed using Student’s *t*-test or two-way ANOVA using GraphPad Prism. *P* < 0.05 was considered statistically significant.

## Results

### Expression Levels of OPG Were Increased in GDM Patients

**FIGURE 1:**
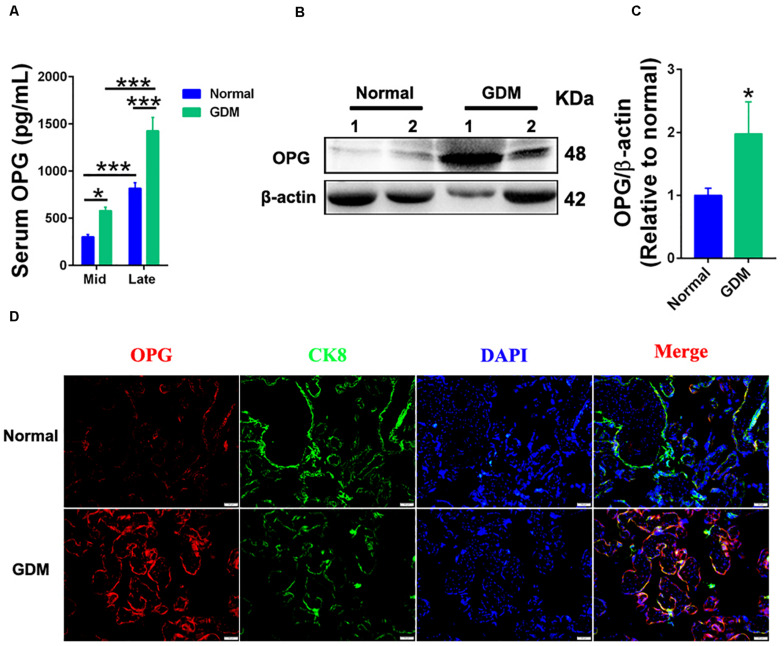
Expression levels of OPG were increased in GDM patients. **(A)** ELISA analysis of serum OPG levels in normal pregnant women and GDM patients in the second (Mid) and late trimesters (Mid: *n* = 16 normal and 17 GDM samples, Late: *n* = 16 normal and 21 GDM samples). **(B)** Representative western blotting analysis of OPG protein expression and **(C)** quantification of OPG protein from panel **(B)** in term placentas of normal pregnant women (*n* = 4) and GDM patients (*n* = 4). **(D)** The colocalization of OPG and CK8 was analyzed in term placentas of normal pregnant women and GDM patients using fluorescence. CK8 is a marker of trophoblasts in placenta, Scale bar = 50 μm. Values are presented as the means ± SEM.*P < 0.05, ***P < 0.001 compared with normal pregnant women.

To investigate the role of OPG on GDM development, we performed ELISA, western blotting and immunofluorescence using normal and GDM samples. As shown in Figure 1A, ELISA results showed significantly increased OPG levels in the GDM patients compared to the normal controls in the middle and late trimesters for both groups. The serum OPG levels measured in the late trimester were significantly higher than those in the middle trimester in both groups. Immunofluorescence revealed that OPG was also expressed in human placental tissues in the third trimester of pregnancy. Western blot analysis showed significantly increased expression levels of OPG in GDM placentas compared to normal term placenta (Figures 1B,C). Similarly, the colocalization of OPG and the trophoblast marker CK8 showed stronger expression in trophoblasts in the placenta of GDM patients than in the normal pregnant women (Figure 1D), suggesting that OPG is involved in the regulation of glucose metabolism homeostasis during pregnancy.

### Upregulation of OPG in Mice During Pregnancy

**FIGURE 2:**
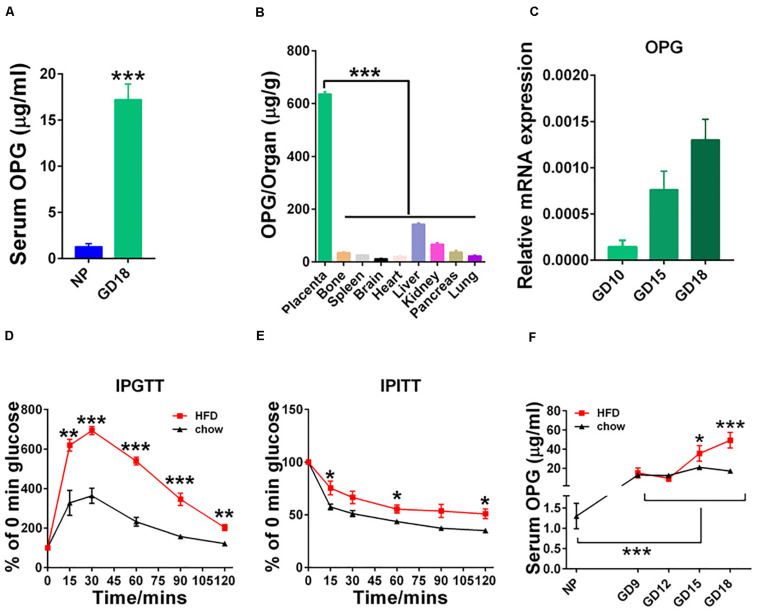
Expression levels of OPG were increased during pregnancy. **(A)** ELISA analysis of serum OPG levels in non-pregnant (NP, *n* = 3) and pregnant (GD18, *n* = 4) mice on day 18. **(B)** ELISA analysis of the expression levels of OPG in different organs in pregnant mice on GD18 (*n* = 4). **(C)** Real-time-PCR analysis of OPG mRNA expression levels in the placentas of chow-fed pregnant mice on GD10, GD15, and GD18 (*n* = 4). **(D)** Intraperitoneal glucose tolerance test (IPGTT) and **(E)** intraperitoneal insulin tolerance test (IPITT) were performed in chow- and HFD-fed pregnant mice on GD16 (*n* = 6). **(F)** ELISA analysis of serum OPG levels in chow- and HFD-fed pregnant mice at non-pregnant (NP), GD10, GD15, and GD18 (4). Values are presented as the means ± SEM. *P < 0.05, **P < 0.01, and ***P < 0.001 compared with NP, placenta and chow, respectively.

To understand the pathophysiological significance of OPG in GDM, we examined the expression of OPG in mice. found that serum OPG levels were significantly increased in pregnant mice compared to non-pregnant mice (Figure 2A). OPG expression levels were analyzed in different tissues, including placenta, bone, spleen, brain, heart, liver, kidney, pancreas and lung, in GD18 mice using ELISA. The placenta tissues showed significantly higher OPG expression than the other tissue types (Figure 2B). Mouse placental expression patterns of OPG using RT-PCR found an increase from GD10 to GD18 during pregnancy (Figure 2C). As shown in Figures 2D,E, we generated the GDM mouse model by feeding the mice HFD, which caused a remarkably increased glucose intolerance and insulin resistance in the IPGTT and IPITT, respectively. This HFD-induced mouse model was reported and used previously (Huang et al., 2019), which indicated that HFD dams have elevated fasting-glucose and body weight has no difference for mice treatment with HFD during pregnancy. The monitoring of serum OPG levels revealed a continuous increase from GD12 to GD18 during pregnancy (Figure 2F). A notable increase in serum OPG levels was found in HFD-fed compared to chow-fed pregnant mice from GD15 to GD18. These results indicated that OPG expression was significantly higher in maternal mice, with especially high expression in the placenta. In conjunction with the finding that OPG was also highly expressed in the HFD-induced GDM mice, these results suggested that OPG played a role in metabolism homeostasis during pregnancy.

### pl-OPG Knockdown Induced Glucose Intolerance in Maternal Mice

**FIGURE 3:**
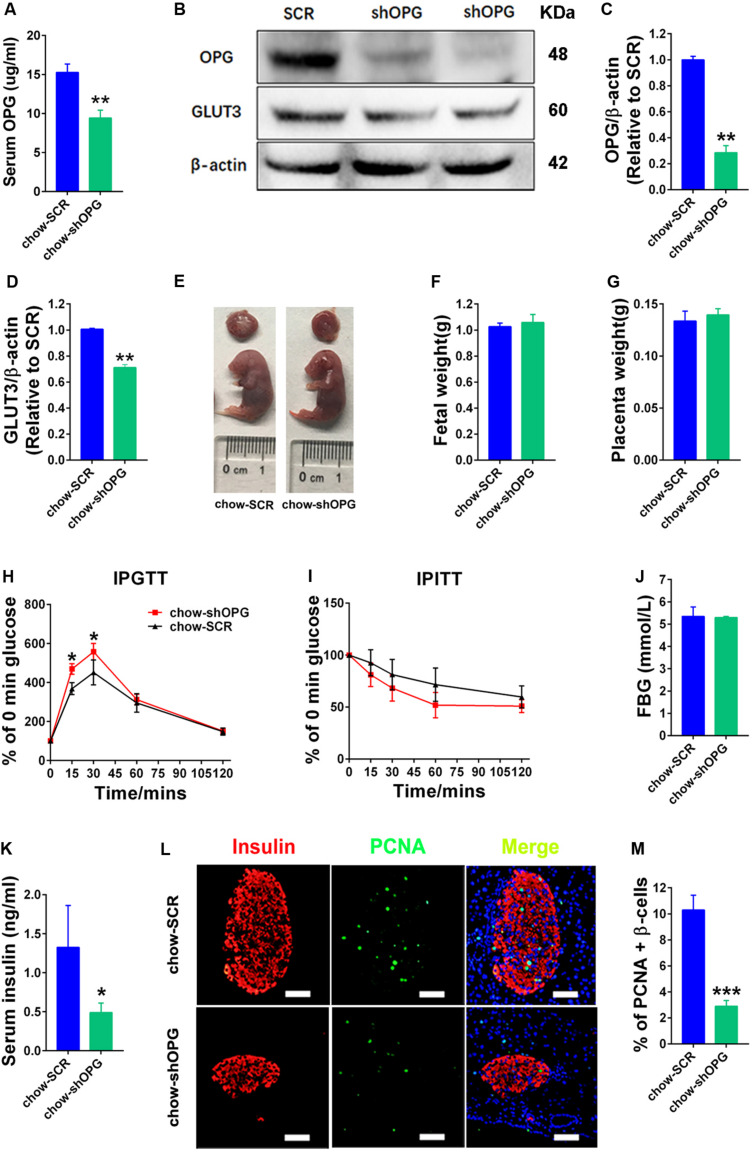
pl-OPG knockdown induced glucose intolerance in maternal mice. **(A)** ELISA analysis showing decreased serum OPG levels in maternal mice with placenta-specific OPG knockdown on GD18 (chow-SCR: *n* = 3, chow-shOPG: *n* = 4). **(B)** Representative Western blotting showing decreased OPG levels in placentas with decreasing GLUT3 levels after transducing with lentiviruses shOPG on GD18. Quantification of OPG **(C)** and GLUT3 **(D)** from panel **(B)**. **(E–G)** pl-OPG shOPG did not significantly alter fetal weight **(F)** or placental weight **(G)** on GD18 (chow-SCR: *n* = 3, chow-shOPG: *n* = 4). **(H)** IPGTT was significantly increased at 15 and 30 min (chow-SCR: *n* = 3, chow-shOPG: *n* = 4). **(I)** IPITT was not different in chow-fed pregnant mice with placenta OPG knockdown on GD16 (chow-SCR: *n* = 3, chow-shOPG: *n* = 4). **(J)** Fasting 6-h blood glucose (FBG) was not significantly different (chow-SCR: *n* = 3, chow-shOPG: *n* = 4). **(K)** Serum insulin levels were significantly decreased in chow-fed pregnant mice with placenta OPG knockdown on GD18 (chow-SCR: *n* = 3, chow-shOPG: *n* = 4). **(L)** Immunofluorescent staining showing **(M)** significant numbers of PCNA-positive β-cells in chow-fed pregnant mice with placenta OPG knockdown on GD18, scale bar = 50 μm. Values are presented as the means ± SEM. *P < 0.05, **P < 0.01, and ***P < 0.001 compared to Scramble (SCR).

To verify the physiological and biological functions of OPG in metabolism homeostasis in maternal mice, we generated placenta-specific OPG knockdown mice fed a chow diet using a previously described method of transducing blastocysts with lentiviruses for trophoblast-specific transgene expression, ELISA and western blotting techniques (Fan et al., 2014). OPG expression levels were significantly decreased in serum (ELISA) (Figure 3A) and placenta (western blot) (Figures 3B,C) in the placenta-specific OPG knockdown mice compared to the SCR group of chow-fed mice. GLUT3 protein expression decreased significantly in the placentas with decreased OPG levels (Figures 3B,D). Fetal and placenta weights were not significantly different between the OPG-knockdown and SCR placenta groups (Figures 3E–G).

We evaluated the effects of OPG on glucose homeostasis in pregnant mice. As predicted by our hypothesis, maternal mice with placental OPG knockdown exhibited significantly impaired glucose tolerance compared to the chow-fed SCR group (Figure 3H). However, the placental OPG knockdown maternal mice exhibited similar insulin tolerance compared to the chow-fed SCR group (Figure 3I). Maternal mice from the placental OPG knockdown group exhibited no significant difference in FBG compared with the SCR group of chow-fed mice (Figure 3J). Insulin secreted by β-cells in the pancreas regulates blood glucose. Our results showed that serum insulin levels dramatically decreased in placental OPG knockdown maternal mice compared to the chow-fed SCR mice (Figure 3K). We also analyzed a marker for proliferation and expression signaling, PCNA, and found a notable decrease in the percentage of PCNA-positive β-cells, which was consistent with immunofluorescence results of colocalization of PCNA and insulin β-cells (Figures 3L,M).

Collectively, these data strongly indicate that placenta-specific OPG plays a role in regulating glucose homeostasis by promoting glucose tolerance, decreasing blood glucose, and increasing insulin secretion. These results suggested that OPG had an important function in glucose metabolism during pregnancy and is required for metabolism homeostasis because insulin production and the proliferation of β-cells during pregnancy required OPG signaling.

### pl-OPG Overexpression Improved Glucose Homeostasis in HFD-Induced GDM Mice

**FIGURE 4:**
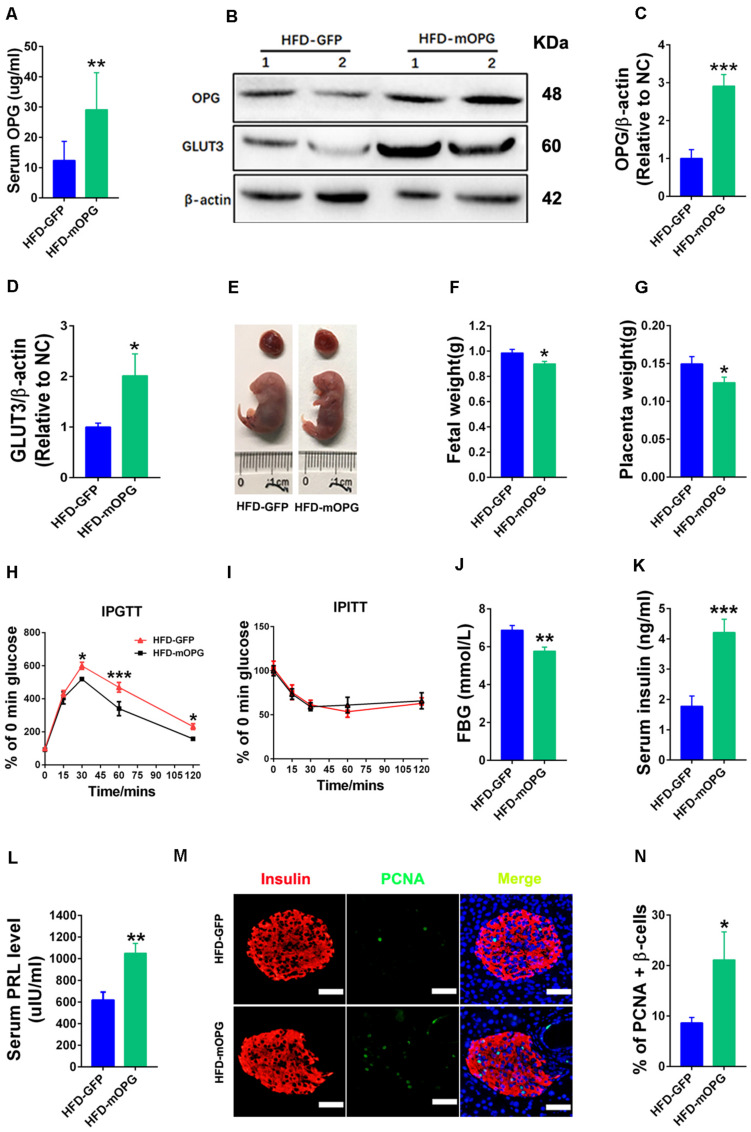
Placenta OPG overexpression improved glucose homeostasis in maternal mice. **(A)** ELISA analysis showing increased serum OPG levels in maternal mice with placenta-specific OPG overexpression on GD18 (HFD-GFP: *n* = 5, HFD-mOPG: *n* = 6). **(B)** Representative western blot showing increased OPG levels in placentas with increasing GLUT3 levels after transducing with lentiviruses mOPG on GD18. Quantification of OPG **(C)** and GLUT3 **(D)** from panel **(B)**. **(E–G)** OPG overexpression significantly decreased fetal weight **(F)** and placental weight **(G)** on GD18 (HFD-GFP: *n* = 5, HFD-mOPG: *n* = 6). **(H)** IPGTT was significantly decreased at 30, 60, and 120 min (HFD-GFP: *n* = 5, HFD-mOPG: *n* = 6). **(I)** IPITT was not different in HFD-fed pregnant mice with placenta OPG overexpression on GD16 (HFD-GFP: *n* = 5, HFD-mOPG: *n* = 6). **(J)** Fasting 6-h blood glucose (FBG) was significantly decreased. **(K)** Serum insulin and **(L)** serum prolactin were significantly increased in HFD-fed pregnant mice with placenta OPG overexpression on GD18 (HFD-GFP: *n* = 5, HFD-mOPG: *n* = 6). **(M)** Immunofluorescent staining showing **(N)** significant numbers of PCNA-positive β-cells in HFD-fed pregnant mice with placenta OPG overexpression on GD18, scale bar = 50 μm. Values are presented as the means ± SEM. *P < 0.05, **P < 0.01, and ***P < 0.001 compared with empty control.

Our current experiments found that placental OPG knockdown impaired glucose tolerance. To further study the developmental role of OPG in GDM, placenta-specific OPG overexpression was established in an HFD-induced GDM mouse model that we previously reported (Huang et al., 2019). Blastocysts were transduced with lentivirus-expressing GFP alone as a control, and a coexpressing OPG overexpression and OPG expression-linked GFP construct was used in the cells with OPG expression. OPG expression levels and fetal and placental development were evaluated. OPG protein expression levels were significantly increased in serum (Figure 4A) and the placenta (Figures 4B,C) in the OPG overexpression mice compared to the control. OPG overexpression induced a significant upregulation of GLUT3 protein in the placentas of these mice (Figures 4B,D). In direct support of previous data showing increased fetal weight in an HFD-induced GDM mouse model (Huang et al., 2019), Figure 4E indicates that pl-OPG improved fetal development and reduced fetal size. Overall, fetal weights were prominently decreased in placenta-specific OPG overexpression mice fed an HFD (Figure 4F). The placenta weight in placenta OPG overexpression mice showed a significant decrease (Figure 4G).

Glucose homeostasis was also evaluated in maternal mice fed an HFD after blastocyst transfer using IPGTT, IPITT, and fasting 6-h blood glucose (FBG). Glucose tolerance was dramatically increased, and blood glucose was significantly decreased at the 30-, 60-, and 120-min time points after glucose injection in the IPGTT in placenta-specific OPG overexpression mice fed an HFD (Figure 4H). However, no difference in insulin sensitivity was observed in the IPITT in the placenta-specific OPG overexpression mice fed an HFD (Figure 4I). We also found a significant decrease in the fasting 6-h blood glucose in the placenta-specific OPG overexpression mice fed an HFD (Figure 4J). ELISA found a significant increase in the serum insulin levels of the placenta-specific OPG overexpression mice compared to control mice fed an HFD (Figure 4K). Consistent with previous reports that prolactin improved maternal β-cell proliferation and insulin secretion during pregnancy (Huang et al., 2009), we also detected significantly increased prolactin levels in placenta-specific OPG overexpression mice compared to the controls fed an HFD (Figure 4L). Therefore, we investigated whether increased OPG enhanced β-cell function and lead to a rescue of GDM via simulating a physiological compensation due to placenta-specific OPG overexpression. We evaluated proliferation using the colocalization of insulin and PCNA in β-cells of maternal mice fed an HFD after blastocyst transfer. The percentage of PCNA-positive β-cells increased notably, which suggests that β-cells had significantly higher proliferation compared to the control (Figures 4M,N).

Taken together, these data showed that placenta-specific OPG overexpression rescued GDM, including improvements in fetal and weight development via decreasing fetal weight and increasing GLUT3 expression in the placenta and restored glucose homeostasis via enhancing β-cell proliferation and insulin production. These results suggest that OPG promotes metabolic activity via regulation of insulin levels and the transport of nutrients in the placenta during pregnancy.

### OPG Directly Promoted β-Cell Proliferation in Maternal Mice

**FIGURE 5:**
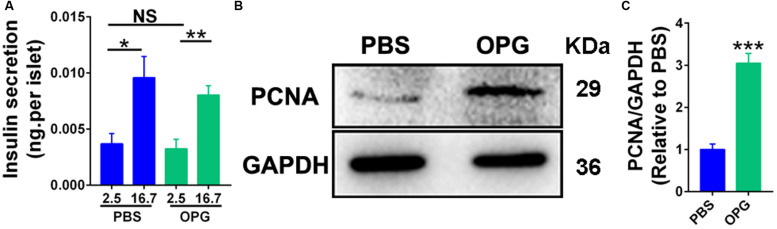
Placenta OPG overexpression promoted β-cell function in HFD-fed mice. **(A)** Islets were treated with 100 ng/mL mouse recombination OPG without FBS for 24 h. GSIS was performed in islets that were preincubated in glucose-free Krebs-Ringer buffer for 1 h and incubated for 1 h in KRB containing glucose (2.5 or 16.7 mM). Insulin secreted into the KRB and insulin contents were measured using ELISA (Millipore). Insulin levels were normalized to the number of islets. **(B)** Representative western blot showing increased PCNA levels in maternal GD16 islets treated with OPG. **(C)** Quantification of PCNA from panel **(B)**. Values are presented as the means ± SEM. *n* = 4, *P < 0.05, **P < 0.01, and ***P < 0.001 compared with control, PBS or low concentrations of glucose (2.5 mM), respectively.

The above results indicated that OPG regulated insulin production during pregnancy. To understand whether OPG stimulated insulin secretion and β-cell proliferation, islets were isolated from maternal mice and treated with mouse OPG. GSIS was performed on primary islets isolated from maternal mice to study the effects of OPG on insulin secretion *in vitro*. Insulin secretion levels were only significantly increased in high concentrations of glucose versus low, and insignificant differences were found between the treated OPG islets and the control (PBS) islets under high and low concentrations of glucose (Figure 5A). This result indicated that OPG does not directly promote insulin secretion in maternal islet. However, PCNA was significantly increased in the maternal islets treated with OPG compared to PBS (Figures 5B,C). This result indicates that OPG promotes the proliferation of maternal islet. OPG also promoted serum prolactin secretion (Figure 4L). Taken together with previous reports that prolactin improved insulin secretion in maternal β-cells (Huang et al., 2009), our results strongly suggest that OPG promotes insulin production via its direct effects on increasing β-cell proliferation and stimulating prolactin secretion in maternal mice.

### OPG Directly Promoted GLUT3 Upregulation in Trophoblasts

**FIGURE 6:**
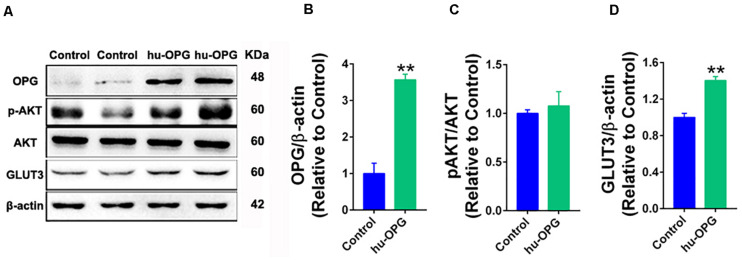
OPG regulated GLUT3 upregulation in trophoblasts. **(A)** Western blotting analysis of OPG, phosphorylated (p)AKT/AKT and GLUT3 expression in HTR-8/SVneo cells. **(B–D)** Quantification of protein expression from panel **(A)**. Three independent experiments were performed. Values are presented as the means ± SEM. **P < 0.01 compared with control.

We generated stable human OPG overexpression HTR-8/SVneo cells to further investigate the OPG pathway in glucose homeostasis in trophoblasts. As shown in Figure 6, OPG protein levels were significantly increased in the OPG-overexpression cell line (Figures 6A,B). GLUT3 also showed a notable increase with OPG upregulation (Figures 6A,D). Phosphorylation (p)AKT/AKT signaling showed no significant difference (Figures 6A,C). These results indicate that OPG may directly regulate GLUT3 expression because phosphorylation (p)AKT/AKT signaling did not change in OPG-overexpression trophoblasts.

## Discussion

**FIGURE 7:**
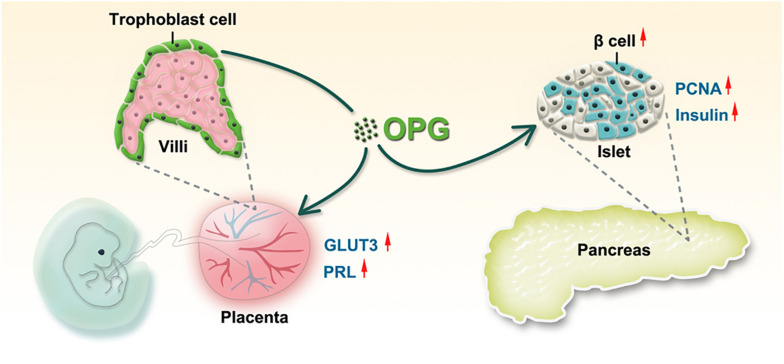
The working model of OPG regulation of glucose homeostasis during pregnancy. OPG directly enhances placental GLUT3 and PRL expression. Placenta-derived OPG stimulates the pancreas to increase islet proliferation and insulin secretion.

We first identified the association of OPG with GDM in clinical samples and found significant increases in OPG levels in pregnant mice in the middle and late trimesters compared to normal mice. As shown in Figure 7, we confirmed that OPG derived and secreted from the placenta was involved in metabolic homeostasis in maternal mice. Placenta-specific OPG knockdown mice given a chow diet exhibited significant glucose intolerance, but placenta-specific OPG overexpression maternal mice fed an HFD exhibited significantly rescued glucose homeostasis. Mechanistically, pl-OPG directly promoted islet β-cell proliferation, which increased serum insulin production in maternal mice. Our current study found that OPG stimulated prolactin production, which was previously reported to improve maternal β-cell proliferation and insulin secretion during pregnancy (Huang et al., 2009). We used placenta-specific OPG overexpression in a GDM model and revealed that OPG derived from placenta acted as a curial regulator in promoting the interaction between endocrine cells in the pancreas and placenta to regulate metabolic homeostasis.

Previous studies showed that OPG played a role in pregnancy complications. OPG levels increased with gestational age growth, with median OPG concentrations being significantly higher in the third trimester than early pregnancy (Hong et al., 2005). OPG was also higher in placentas of preeclampsia compared to normal pregnancy at the protein and mRNA levels (Shen et al., 2012). The current study found that serum OPG levels were dramatically increased in GDM patients compared to normal pregnant women. OPG levels were also increased in the HFD-induced GDM mouse model, as previously reported (Huang et al., 2019). These studies reveal the potential role of OPG in pregnancy complications, particularly metabolic disorders during pregnancy. Several conflicting studies indicated that OPG induced normal or impaired glucose tolerance in mice. Kondegowda et al. (2015) revealed that OPG acted as a β-cell mitogen and enhanced β-cell proliferation in young, aged, and diabetic mice via lactogen mediation. In contrast, Toffoli et al. (2011) reported that mice injected with human recombinant OPG at low doses (1.0 μg/mouse) showed islet inflammation and β-cell death, which led to hypoinsulinemia and hyperglycemia. However, human OPG generated antibodies in rats against the human peptide, which may explain these negative effects (Mochizuki et al., 2002).

Collectively, OPG was increased in the placentas of GDM patients compared to normal pregnant women. Notably, serum OPG levels in mice were also increased during pregnancy, with the highest expression found in the placenta. Previous studies revealed that a systemic increase of OPG using transgenic overexpression of OPG in rats did not regulate blood glucose homeostasis in the long term (Ominsky et al., 2009). Given the short gestation period in mice, we generated placenta-specific OPG expression changes to study whether OPG was involved in metabolism homeostasis in maternal mice, as previously reported (Fan et al., 2014). Based on our findings, placental OPG knockdown impaired glucose homeostasis (Figure 3). As previously shown, macrosomia is one of the risks of GDM development (Demirci et al., 2012; Qiao et al., 2015), which supports our previous result of a significant increase in the fetal weights in the HFD-induced GDM mice model (Huang et al., 2019). Fetal weight was decreased in mice with placenta-specific overexpression of OPG in our study. GLUT3 protein expression correlated with OPG levels in maternal mice, with decreased GLUT3 in the placenta-specific OPG knockdown group fed a chow diet and increased GLUT3 in the OPG-overexpression group fed an HFD, which may impact glucose transport and fetal growth during pregnancy (Brown et al., 2011; Janzen et al., 2013). GPR1 may regulate GLUT3 expression via phosphorylation (p)AKT/AKT signaling, as previously described (Huang et al., 2019). Mechanistically, OPG did not change phosphorylation (p)AKT/AKT signaling, which suggests that OPG directly regulates GLUT3 expression *in vitro*. These results indicate that pl-OPG majorly improved the symptoms of GDM. Consistent with the results that OPG regulated blood glucose levels in GDM, with the knockdown and overexpression models showing opposite effects, the tested insulin levels were increased in maternal mice with placenta-specific overexpression of OPG fed an HFD. PCNA-positive β-cells were also increased in maternal mice with OPG overexpression, which suggests that OPG promotes maternal β-cell proliferation. The evaluated prolactin levels were increased in maternal mice with OPG overexpression in the current study, which may also directly increase β-cell proliferation and insulin secretion (Brelje et al., 2004; Demirci et al., 2012). However, we only found that OPG led to maternal mice β-cell proliferation, not direct stimulation of insulin secretion *in vitro*, which suggests that OPG directly stimulates β-cell proliferation and promotes insulin secretion via prolactin in maternal mice in GDM status.

## Conclusion

In conclusion, we found a novel feedback mechanism related to the regulation of glucose homeostasis during pregnancy.

## Data Availability Statement

The original contributions presented in the study are included in the article/supplementary material, further inquiries can be directed to the corresponding author/s.

## Ethics Statement

The studies involving human participants were reviewed and approved by Committee on the Use of Humans for Teaching and Research, Shenzhen Institutes of Advanced Technology, Chinese Academy of Sciences. The patients/participants provided their written informed consent to participate in this study. The animal study was reviewed and approved by Committee on the Use of Live Animals for Teaching and Research, Shenzhen Institutes of Advanced Technology, Chinese Academy of Sciences.

## Author Contributions

JZ designed this study. BH and FZ contributed to the clinic research data. BH and HZ wrote this manuscript. JZ, BH, JN, and LR contributed the research design. BH, WZ, HZ, JC, and ML conducted the animal and cell experiments. EW, HZ, CH, and HW revised and edited this manuscript. JZ was the guarantor of this work and, as such, had full access to all the data in the study and takes responsibility for the integrity of the data and the accuracy of the data analysis. All authors contributed to the article and approved the submitted version.

## Conflict of Interest

The authors declare that the research was conducted in the absence of any commercial or financial relationships that could be construed as a potential conflict of interest.

## Abbreviations

FBG: fasting blood glucose
GDM: gestational diabetes mellitus
GLUT3: glucose transport protein 3
GPR1: G protein-coupled receptor 1
GSIS: glucose-stimulated insulin secretion
HFD: high-fat diet
IPGTT: Intraperitoneal Glucose Tolerance Test
NC: negative control
OGTT: oral glucose tolerance test
OPG: osteoprotegerin
pAKT: AKT phosphorylation
PCNA: proliferating cell nuclear antigen
pl-OPG: placenta-derived OPG
PRL: prolactin
RIA: radioimmunoassay.
